# *dyschronic*, a *Drosophila* Homolog of a Deaf-Blindness Gene, Regulates Circadian Output and Slowpoke Channels

**DOI:** 10.1371/journal.pgen.1002671

**Published:** 2012-04-19

**Authors:** James E. C. Jepson, Mohammad Shahidullah, Angelique Lamaze, Drew Peterson, Huihui Pan, Kyunghee Koh

**Affiliations:** Department of Neuroscience, Farber Institute of Neuroscience, Kimmel Cancer Center, Thomas Jefferson University, Philadelphia, Pennsylvania, United States of America; University of California San Francisco, United States of America

## Abstract

Many aspects of behavior and physiology are under circadian control. In *Drosophila*, the molecular clock that regulates rhythmic patterns of behavior has been extensively characterized. In contrast, genetic loci involved in linking the clock to alterations in motor activity have remained elusive. In a forward-genetic screen, we uncovered a new component of the circadian output pathway, which we have termed *dyschronic* (*dysc*). *dysc* mutants exhibit arrhythmic locomotor behavior, yet their eclosion rhythms are normal and clock protein cycling remains intact. Intriguingly, *dysc* is the closest *Drosophila* homolog of *whirlin*, a gene linked to type II Usher syndrome, the leading cause of deaf-blindness in humans. Whirlin and other Usher proteins are expressed in the mammalian central nervous system, yet their function in the CNS has not been investigated. We show that DYSC is expressed in major neuronal tracts and regulates expression of the calcium-activated potassium channel SLOWPOKE (SLO), an ion channel also required in the circadian output pathway. SLO and DYSC are co-localized in the brain and control each other's expression post-transcriptionally. Co-immunoprecipitation experiments demonstrate they form a complex, suggesting they regulate each other through protein–protein interaction. Furthermore, electrophysiological recordings of neurons in the adult brain show that SLO-dependent currents are greatly reduced in *dysc* mutants. Our work identifies a *Drosophila* homolog of a deaf-blindness gene as a new component of the circadian output pathway and an important regulator of ion channel expression, and suggests novel roles for Usher proteins in the mammalian nervous system.

## Author Summary

In most organisms, endogenous circadian clocks help to restrict adaptive activities such as foraging and mating to ecologically appropriate periods of the day–night cycle. The fruit fly *Drosophila melanogaster* has been a crucial genetic model system for understanding the molecular underpinnings of the clock. Here, using a forward-genetic screen for mutant flies that lack circadian patterns of locomotion, we identify a novel gene critical to circadian behavior, which we have termed *dyschronic* (*dysc*). Interestingly, DYSC is not part of the molecular clock itself, but acts in an intermediate circuit between clock cells and motor neurons to regulate temporal alterations in locomotion. DYSC contains several protein-binding domains, suggesting a role as a scaffolding protein. Indeed, we show that DYSC forms a mutually dependent complex with the SLOWPOKE Ca^2+^–activated potassium channel, an ion channel required for circadian output. DYSC regulates SLOWPOKE expression and SLOWPOKE-dependent currents in the fly brain. Furthermore, *dysc* is the closest *Drosophila* homolog of *whirlin*, a locus mutated in the human deaf-blindness disease Type II Usher syndrome. Our results identify a novel ion channel regulator that impacts neuronal physiology and complex behavior, and suggest new roles for Whirlin in the human nervous system.

## Introduction

In diverse phyla, circadian systems act to synchronize changes in arousal and internal physiology to optimal time periods for feeding, courtship, and other ethologically relevant behaviors. In *Drosophila*, the molecular basis of the internal clock that drives such rhythmic alterations in behavior has been extensively characterized [1]. Molecular and genetic approaches have demonstrated that a transcriptional negative-feedback loop lies at the heart of the clock, in which the transcription factors CLOCK and CYCLE activate expression of their own repressors, PERIOD (PER) and TIMELESS [1]. In combination with additional modulatory feedback loops and post-translational regulatory mechanisms, oscillatory activation of CLOCK/CYCLE leads to temporally controlled expression of a wide range of clock-controlled genes, thus altering the functional properties of clock neurons in a time-dependent manner [2]–[6].

In contrast to the core clock mechanism, only a small number of genes that act downstream of the clock have been identified, including *pigment dispersing factor* (*pdf*), *pdf receptor* (*pdfr*), *neurofibromatosis-1* (*nf1*), *slowpoke* (*slo*), *narrow abdomen* (*na*), and *ebony* [7]–[14]. Two of these output genes encode voltage-gated ion channels, SLO and NA, suggesting that modulation of neuronal excitability is an essential component of circadian output. Of the two channels, the electrophysiological properties and cellular consequences of SLO channels have been defined in much greater detail. SLO is a member of the BK (big K^+^) family of voltage-gated Ca^2+^-activated potassium channels, and generates non-inactivating K^+^ currents with high single-channel conductance [15], [16]. SLO channels act to repolarize the membrane potential during action potentials, and *Drosophila slo* mutants thus exhibit broader action potentials in flight muscles and cultured neurons [17]–[19]. Intriguingly, BK channel function is critical for circadian behavior in both *Drosophila* and mammals. *Drosophila slo* mutants are arrhythmic, yet restoring SLO expression in clock neurons does not robustly rescue rhythmic behavior, suggesting that SLO acts downstream of clock cells [2], [7]. Mammalian BK channels are also required for clock output from the suprachiasmatic nucleus (SCN), and contribute to the silencing of SCN neurons during the night [20].

Consistent with the key role of ion channels in the control of neuronal physiology and behavior, regulators of ion channel function have also been found to modulate behavioral outputs. For example, SLEEPLESS, a positive regulator of Shaker potassium channels, strongly affects sleep in *Drosophila* [21]. Here we identify a novel SLO-binding protein, which we have termed DYSCHRONIC (DYSC). *dysc* mutants exhibit arrhythmic locomotor activity but normal eclosion rhythms and wild-type molecular oscillations in clock neurons, suggesting *dysc* is specifically required for circadian locomotor output. Intriguingly, DYSC is the closest *Drosophila* homolog of Whirlin, a PDZ (PSD-95/DLG/ZO-1) domain-containing protein mutated in Type II Usher syndrome, a human deaf-blindness disease [22], [23]. Through targeted rescue experiments, we demonstrate that DYSC acts downstream of clock cells to control locomotor output. We show that DYSC co-localizes with SLO in major neuronal tracts in the brain, and that the two proteins form a complex to regulate each other's expression post-transcriptionally. Furthermore, SLO-dependent potassium currents are significantly reduced in *dysc* neurons in the adult brain. Our results define a novel channel regulator required for rhythmic alterations in behavior and suggest new roles for Whirlin in the mammalian brain.

## Results

### *dyschronic* Is Required for Circadian Locomotor Output in *Drosophila*

**Figure 1:**
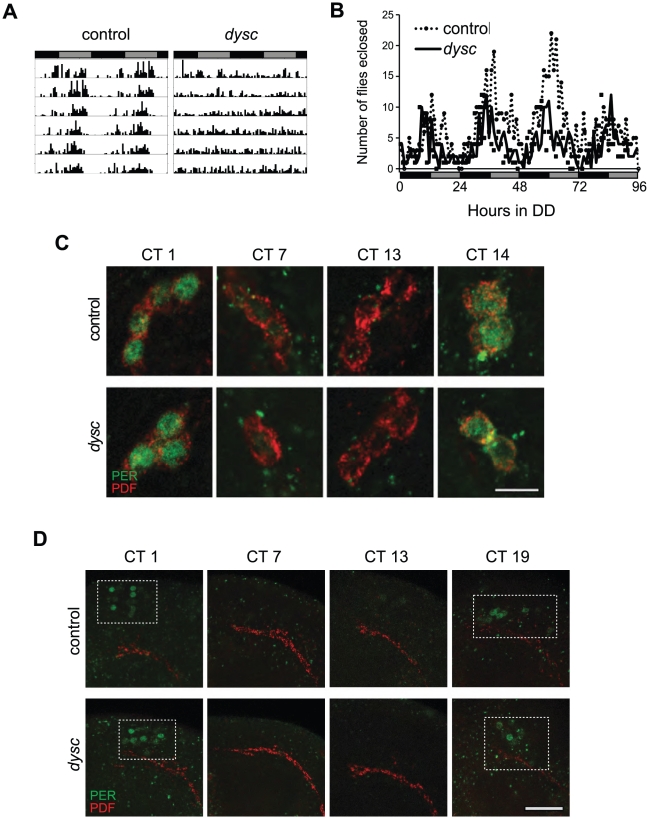
The *dysc* mutation defines a novel circadian output gene.

In an ongoing forward-genetic screen for sleep and circadian mutants, we identified an arrhythmic mutant line resulting from a P-element insertion, which we named *dyschronic* ^s168^ (*dysc* ^s168^). Most *dysc* ^s168^ homozygotes were arrhythmic in constant-dark (DD) conditions, with a minority showing weak rhythmicity (Figure 1A and Table 1). To assess whether *dysc* regulates circadian behavior in general or locomotor behavior specifically, we examined circadian patterns of eclosion from the pupal case, a behavior that is dependent on correct output from small ventral lateral neurons (s-LN_v_s), a subset of clock neurons required for rhythmic locomotion in DD [12], [24]. Interestingly, despite clear arrhythmic locomotor patterns in *dysc* mutants, we observed an eclosion rhythm that closely mirrored wild-type controls (Figure 1B), suggesting that s-LN_v_ output is unimpaired in *dysc* mutants.

**Table 1:** Free-running circadian locomotor rhythm phenotypes in DD.

Genotype	*N* a	% R^a^	% WR^a^	% AR^a^	Tau (h) ± SEM^b^	Power ± SEM^b^
control^c^	99	97	3.0	0	23.63±.04	117.0±3.3
*dysc* ^s168^	36	0	5.6	94.4	nd^d^	−1.5±2.6
*dysc* ^c03838^	36	0	13.9	86.1	nd	11.7±3.1
*dysc* ^c05107^	42	47.6	16.7	35.7	24.0±.12	48.2±5.3
*dysc* ^s168^/+	30	100	0	0	23.45±.03	121.6±5.3
*dysc* ^c03838^/+	32	93.8	3.1	3.1	23.5±.07	107.1±6.2
*dysc* ^s168/c03838^	24	8.3	20.8	70.8	24.5±1	13.1±5.2
*dysc* ^s168/c05107^	30	50	16.7	33.3	23.6±.09	47.2±5.6
*dysc* ^c03838/c05107^	28	32.1	17.9	50	23.6±.11	37.4±7.7
*dysc* ^Df[BSC614]^ /+	23	100	0	0	24.0±.08	117.5±4.8
*dysc* ^s168/Df[BSC614]^	22	13.6	27.3	59.1	24.0±.29	17.1±5.5
*dysc* ^c03838/Df[BSC614]^	29	10.3	17.2	72.4	24±.5	20.6±6.1
*dysc* ^pr9^ e	29	96.6	3.5	0	23.6±.06	126.9±6.0

^**a**^ *N*: number of flies; R: rhythmic; WR: weakly rhythmic; AR, arrhythmic.

^**b**^ *χ^2^* periodogram analysis was performed for each fly using the FassX software to determine the free-running period, tau. Power, a measure of rhythmicity, corresponds to peak – significance value (at *p* = 0.05).

^**c**^Three controls strains, one for each of the *dysc* mutant alleles, produced comparable results, and pooled data are presented.

^**d**^nd: not determined.

^**e**^pr9: Precise excision line derived from s168.

To determine whether DYSC functions as a component of the clock, we next assessed the integrity of molecular oscillations in clock neurons in *dysc* mutants. We examined PER cycling in two sets of clock neurons: s-LN_v_s and a cluster of dorsal neurons (DN1s), which has been proposed to be a direct target of output from the s-LN_v_s [25]. In *dysc* mutants, we found that daily cycling of PER expression was indistinguishable from wild-type controls in both sets of clock neurons (Figure 1C, 1D). Similarly, PER levels exhibited wild-type oscillatory patterns in head extracts of *dysc* mutants (Figure S1). Since the bulk of PER protein in head extracts derives from eye tissue, this suggests that the molecular clock is unimpaired in the periphery as well. These findings establish that core clock function is normal in *dysc* mutants, and thus identify DYSC as a constituent of the circadian locomotor output pathway.

### *dysc* Is a *Drosophila* Homolog of *whirlin*, a Human Deaf-Blindness Gene

**Figure 2:**
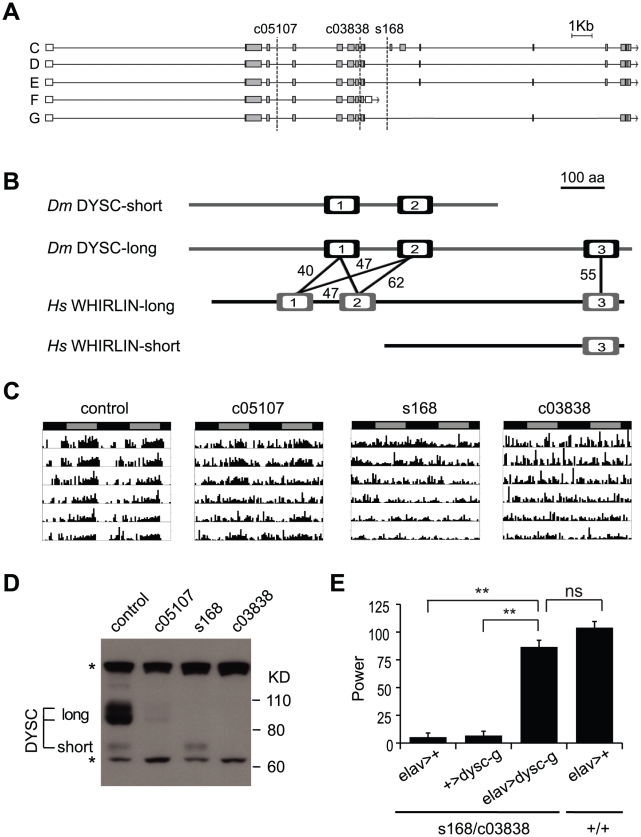
*dysc* is the *Drosophila* ortholog of *whirlin*, a member of the mammalian Usher complex. (A) Schematic illustrating differentially spliced isoforms arising from the *dysc* transcription unit, and the insertion sites (dotted lines) of the three alleles used in this study. (B) Isoform F yields a shorter isoform containing two PDZ domains (open boxes), while the remaining isoforms contain three PDZ domains. Percentage identity between the PDZ domains of DYSC and human Whirlin are shown. The *whirlin* transcription unit, like *dysc*, generates long and short isoforms, as well as one containing the C-terminal PDZ domain alone. (C) Representative actograms of individual control and *dysc* ^c05107^, *dysc* ^s168^ and *dysc* ^c03838^ homozygous flies in DD. (D) Western blot showing DYSC long and short isoform expression in the above genotypes. MAPK bands are shown for loading control. * non-specific labeling. (E) Pan-neuronal expression by *elav*-*Gal4* of a UAS-*dysc* transgene encoding a long DYSC isoform (Isoform G) in a *dysc* ^s168/c03838^ trans-heterozygotic background increased the power, or strength, of circadian rhythmicity relative to driver-alone and transgene-alone controls to levels statistically indistinguishable from a wild-type control containing the *elav*-*Gal4* driver. See also Table 2. Error bars represent SEM. ** *p*<0.0001; ns: not significant; two-tailed t-test with Bonferroni correction.

We mapped the s168 P-element insertion to an intron in a previously uncharacterized locus, CG34400 (Figure 2A). The *dysc* transcription unit generates two predominant classes of mRNAs via alternative splicing: multiple long isoforms and a short isoform (Figure 2A). Full-length *dysc* transcripts encode proteins containing three PDZ domains (Figure 2B), a motif commonly associated with scaffolding proteins [26], while the short DYSC isoform lacks the C-terminal PDZ domain. Intriguingly, comparative genomics identified *dysc* as the closest *Drosophila* homolog of *whirlin* (*USH2D*, *DFNB31*), a gene implicated in type II Usher syndrome (USH2) in humans [22]. Like *dysc*, the mammalian *whirlin* locus encodes several distinct splice-forms, including those corresponding to long *dysc* isoforms as well as one containing the C-terminal PDZ domain alone [22], [27] (www.ensembl.org; Figure 2B).

USH2 is characterized by early-age hearing loss due to alterations in ear cell stereocilia formation followed by progressive blindness resulting from photoreceptor degeneration [23]. Genetic ablation of *whirlin* in mice also leads to hearing loss coupled with abnormal photoreceptor development [27], [28]. In addition to sensory tissues, Whirlin is also expressed in the mammalian central nervous system [29], [30], yet the function of Whirlin in the brain is unclear. The identification of *dysc* thus provides a platform in a genetically amenable model organism to investigate novel functions of a Whirlin homolog.

### Loss of DYSC Results in Arrhythmic Locomotor Behavior

We performed several independent genetic experiments to establish that specific disruption of the *dysc* locus is indeed causative of the arrhythmic phenotype in *dysc* mutants. We obtained two additional P-element insertions in *dysc* (c03838 and c05107) (Figure 2A). Like *dysc* ^s168^, most *dysc* ^c03838^ mutants were arrhythmic in DD (Figure 2C and Table 1). On the other hand, *dysc* ^c05107^ mutants exhibited a milder circadian phenotype, with some showing robust rhythmicity (Figure 2C and Table 1). To analyze the effects of the three separate P-element insertions on DYSC expression, we generated a polyclonal antibody to DYSC. Part of the DYSC antigen is common to all isoforms, and thus the antibody is expected to recognize all isoforms. As expected, in wild-type adult head extracts, we observed multiple bands corresponding to the predicted long isoforms, and a single band at the expected size of the short isoform (Figure 2D). Western blotting further revealed differential effects of the three P-element insertions on the expression of DYSC isoforms (Figure 2D). The *dysc* ^c05107^ insertion acts as a hypomorphic allele, leaving expression of all DYSC isoforms reduced but still detectable. In contrast, *dysc* ^c03838^ renders expression of all DYSC isoforms undetectable, and is therefore a null or a strong hypomorphic allele. The remaining DYSC expression in *dysc* ^c05107^ homozygotes is thus likely to be sufficient to partially rescue rhythmic behavior. While *dysc* ^s168^ also renders the long isoforms undetectable, it leaves expression of the short isoform intact. Our finding that the s168 mutation causes as strong a circadian phenotype as c03838 suggests that the short isoform is not sufficient for rhythmic behavior and that the C-terminal PDZ domain plays an important role in circadian rhythms.

In addition to assessing rhythmicity in DD conditions, we also examined locomotor patterns of *dysc* mutants in 12 h light∶dark (LD) conditions (Figure S2A). In contrast to wild-type flies, *dysc* ^s168^ and *dysc* ^c03838^ homozygotes did not exhibit anticipation of lights-on, further suggesting that output of the morning oscillator (which drives rhythmic behavior in DD) is impaired by loss of DYSC. Hypomorphic *dysc* ^c05107^ flies showed normal morning anticipation, while anticipation of lights-off was maintained in all *dysc* allelic backgrounds (Figure S2A). Overall daytime and nighttime activity in LD conditions was greater in *dysc* mutants relative to wild type controls (Figure S2B). This is in contrast to climbing defects observed in *dysc* mutants (Figure S2C), and suggests that although *dysc* flies have some motor problems, overall inactivity is not a contributing factor for arrhythmicity.

All *dysc* alleles were recessive; trans-heterozygotic combinations of the three alleles were largely arrhythmic; and heterozygosity for both *dysc* ^s168^ and *dysc* ^c03838^ in combination with a deficiency removing the *dysc* locus also resulted in arrhythmia (Table 1). In addition, precise excision of the *dysc* ^s168^ P-element restored wild-type patterns of locomotion (Table 1), indicating that the P-element insertion is responsible for arrhythmicity. Finally, to test whether transgenic expression of *dysc* could restore rhythmic behavior, we generated flies carrying a UAS-*dysc* transgene encoding a long isoform of DYSC. Pan-neuronal expression of the UAS-*dysc* transgene in *dysc* mutants was sufficient to rescue rhythmic behavior (Figure 2E and Table 2). Consistent with the fact that *dysc* ^s168^ homozygotes, which express normal levels of the short isoform, are arrhythmic, transgenic expression of a short DYSC isoform did not restore rhythmicity in *dysc* ^c03838^ mutants (Table 2; see Figure S3 for expression levels of long and short *dysc* transgenes). Over-expression of either the long or short isoforms of DYSC in a wild-type background did not affect circadian rhythmicity (Table 2). These results comprehensively demonstrate that DYSC is required for circadian alterations in locomotor activity, and furthermore indicate that correct circadian output requires DYSC expression in the nervous system.

**Table 2:** Phenotypic consequences of targeted restoration and over-expression of DYSC.

Genotype	N	% R	% WR	% AR	Tau (h) ± SEM	Power ± SEM
Rescue						
*elav-Gal4*/Y;; *dysc* ^c03838/s168^	34	3.0	14.7	82.4	nd	5.9±3.6
+/Y;UAS-*dysc-g*/+; *dysc* ^c03838/s168^	30	6.7	6.7	86.7	24.8±.25	6.8±3.9
*elav-Gal4*/Y; UAS-*dysc-g*/+; *dysc* ^c03838/s168^	35	85.7	11.4	2.9	23.5±.09	86.7±5.9
+/Y;UAS-*dysc-f*/+; *dysc* ^c03838/s168^	33	3.0	3.0	93.9	nd	−1.8±3.4
*elav-Gal4*/Y; UAS-*dysc-f*/+; *dysc* ^c03838/s168^	25	4.0	16.0	80.0	nd	10.6±4.9
Overexpression						
*elav-Gal4*/Y	49	89.8	6.1	4.1	23.8±.06	98.7±4.7
*+*/Y;UAS-*dysc-g*/+	31	93.5	3.2	3.2	23.7±.07	98.4±6.2
*elav-Gal4*/Y; UAS-*dysc-g*/+	32	84.4	6.3	9.4	23.6±.08	83.3±6.8
*+*/Y;UAS-*dysc-f*/+	31	90.3	3.2	6.5	24.1±.09	90.8±6.4

### DYSC Is Enriched in Major Neuronal Tracts in the *Drosophila* Nervous System

**Figure 3:**
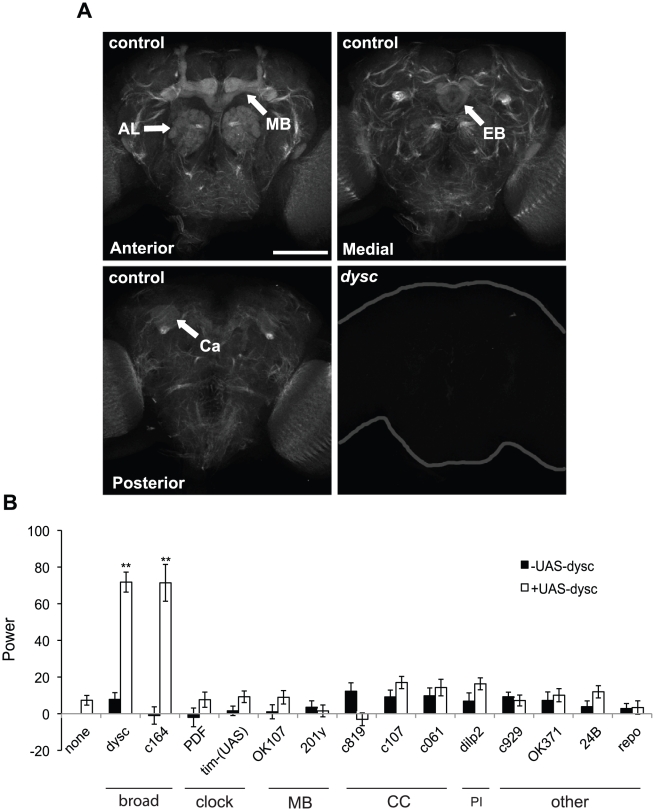
DYSC is enriched in neuronal tracts and is required downstream of the clock cells. (A) Strong DYSC staining was observed in major neuronal tracts throughout the central brain in adult control flies, as well as in the antennal lobes (AL), mushroom bodies (MB) and ellipsoid body (EB). Upper panels show confocal projections spanning the anterior and medial compartments of the adult brain, and lower left panel shows a single 2 µm slice to illustrate DYSC expression in the posterior calyx (Ca) of the mushroom bodies. Similar DYSC immuno-reactivity was not observed in *dysc* ^c03838^ homozygous brains (lower right panel), confirming the specificity of the antibody. Scale bar represents 100 µm. (B) Targeted rescue of the *dysc* circadian phenotype using the UAS-*dysc* transgene (Isoform G). Data are from homozygous *dysc* ^c03838^ or trans-heterozygous *dysc* ^c03838/s168^ flies. In addition to *dysc* mutants carrying a UAS-*dysc* transgene insertion alone, driver-alone controls were used for each driver line. The power of rhythmicity in DD is shown for each genotype (*N*≥21, except for *pdf*-*Gal4* driver control, for which *N* = 17). MB: mushroom bodies; CC: central complex; PI: pars intercerebralis. c929- and OK371-*Gal4* lines drive expression in peptidergic and glutamatergic neurons, respectively. 24B- and *repo*-*Gal4* lines are tissue-specific drivers for muscle and glia, respectively. Error bars represent SEM. ** *p*<0.0001; two-tailed t-test with Bonferroni correction.

We next examined DYSC expression in the adult *Drosophila* nervous system by performing whole-mount immuno-staining of the adult brain. DYSC-specific immuno-reactivity was enriched in major neuronal tracts, i.e., dense bundles of neuronal processes, throughout the central brain (Figure 3A). Interestingly, in the mushroom bodies, ellipsoid body and antennal lobes, DYSC expression was broader than in other regions. For example, DYSC expression was observed throughout the mushroom body including the lobes, peduncle, and calyx, although not in the cell bodies (Figure 3A). In fact, no cell-body expression of DYSC was detected in any brain region. To define the cell-body locations of DYSC-expressing neurons, we generated transgenic flies carrying *Gal4* under the control of the *dysc* promoter. Consistent with the widespread expression of DYSC, GFP expression driven by *dysc-Gal4* was detected in many cell bodies in the brain (Figure S4A), including subsets of clock neurons (Figure S4B), and expression of DYSC using the *dysc*-*Gal4* driver restored rhythmic behavior in *dysc* mutants (Figure 3B).

To examine whether DYSC expression was under circadian control, as has previously been documented for certain output genes [2], [13], we examined DYSC expression and localization at various circadian time-points. These experiments revealed that DYSC protein levels in head extracts were not subject to circadian cycling, nor was any obvious temporal alteration in DYSC expression and localization in the adult brain observed (Figure S5). However, we cannot rule out the possibility that DYSC undergoes circadian regulation in a subset of cells.

### DYSC Is Required Downstream of the Central Clock for Rhythmic Behavior

We attempted to narrow down the key DYSC-expressing cells required for circadian locomotor behavior using a targeted rescue strategy (Figure 3B). Complementing our pan-neuronal rescue data, transgenic expression of DYSC in muscle or glial cells did not restore rhythmic behavior (Figure 3B). Expression of DYSC in PDF- or TIM-expressing clock neurons also failed to rescue the circadian phenotype (Figure 3B). We next attempted to rescue *dysc* mutant arrhythmicity via targeted expression of UAS-*dysc* to major centers in the *Drosophila* nervous system. Expression of DYSC in the central complex, pars intercerebralis or the mushroom bodies, regions of the *Drosophila* brain implicated in motor control and complex behaviors [31]–[33], was insufficient to restore rhythmicity. Whereas c164-*Gal4*, a driver widely used for expression in motor neurons, robustly rescued circadian behavior, OK371-*Gal4*, which drives expression in glutamatergic neurons, including motor neurons, did not (Figure 3B). c164-*Gal4* drives expression in several brain regions in addition to motor neurons [34] (Figure S6A), but not in the ellipsoid body, a region important for locomotor behavior [32]. Co-staining with PER shows that it also drives expression in a few clock cells (Figure S6B). Given that *dysc*-*Gal4* drives expression in many clock cells (Figure S4B) and that a recent study identified *dysc* as a potential direct target of CLK [35], DYSC may function in both clock and non-clock cells. However, our results clearly show that DYSC expression in clock cells alone is not sufficient to restore rhythmicity. Combined with our data indicating that DYSC does not affect clock protein oscillations (Figure 1), this suggests that DYSC is required downstream of clock neurons.

Collectively, these results indicate a role for DYSC in an intermediary circuit between the central clock neurons and motor neurons, and further suggest that the cellular requirements for DYSC in the circadian output circuit are likely to be structurally complex and not easily recapitulated using restricted driver lines.

### DYSC Is Required for Expression of SLO Channels

In mammalian photoreceptors and cochlear stereocilia, Whirlin, the mammalian DYSC homolog, forms a scaffolding complex to properly localize the transmembrane proteins Usherin and Very large G-protein-coupled receptor 1 (VLGR1) [27], [29], [36], [37]. We hypothesized that DYSC might also be required for appropriate transmembrane protein localization in the *Drosophila* nervous system. Since Usher proteins have previously been shown to interact with ion channels [38], we focused on SLO, a Ca^2+^-activated potassium channel, which is required for clock output in flies and mammals [2], [7], [20].

**Figure 4:**
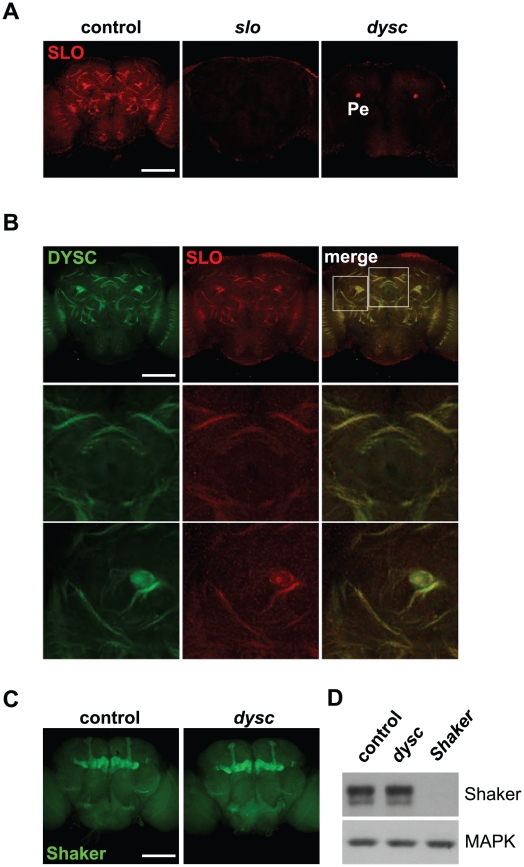
DYSC regulates expression of SLO. (A) Maximum-intensity projections of confocal sections illustrating enrichment of SLO in neuronal tracts in the medial compartment of the central brain of wild-type control (left panel), *slo* ^4^ (middle) and *dysc* ^c03838^ (right) males. SLO staining in neuronal tracts was undetectable in both *slo* ^4^ and *dysc* ^c03838^ mutants. However, we still observed robust SLO immuno-reactivity in the mushroom body peduncle (Pe) of *dysc* ^c03838^ homozygotes. Scale bar represents 100 µm. (B) DYSC and SLO show strong co-localization in the adult brain. Upper panels, 2 µm confocal slice of a medial section of the adult brain. Middle and lower panels show magnified images of regions indicated in the upper right panel. (C–D) Shaker localization and expression is not altered in *dysc* mutants. (C) Confocal projection of Shaker expression in adult control and *dysc* ^c03838^ male brains. (D) Shaker protein expression in head extracts of wild-type control, *dysc* ^c03838^, and *Shaker* ^Df^ flies. MAPK was used as a loading control. The experiment was performed three times with similar results.

Using a new anti-SLO antibody, we observed clear enrichment of SLO in major neuronal tracts throughout the central brain (Figure 4A). SLO staining in neuronal tracts was not detected in *slo* ^4^ mutants (Figure 4A), confirming the specificity of the antibody. Intriguingly, DYSC and SLO exhibited a high degree of co-localization in neuronal tracts (Figure 4B). Unlike DYSC, however, we did not observe strong SLO staining in the mushroom body lobes, calyx or the ellipsoid body (Figure S7). Given the degree of overlapping expression, we examined whether SLO expression was altered in *dysc* mutants. Remarkably, SLO staining in neuronal tracts was undetectable in *dysc* mutants (Figure 4A), and the only remaining SLO signal within the brain was localized to the mushroom body peduncle. To test whether voltage-gated potassium channels in general were affected in *dysc* mutants, we examined the expression and localization of the A-type potassium channel, Shaker. In contrast to SLO, Shaker protein levels and localization within the brain were unaffected in *dysc* flies (Figure 4C–4D). Thus, DYSC specifically regulates the expression of a potassium channel subtype.

### SLO-Dependent Currents Are Markedly Reduced in *dysc* Mutants

**Figure 5:**
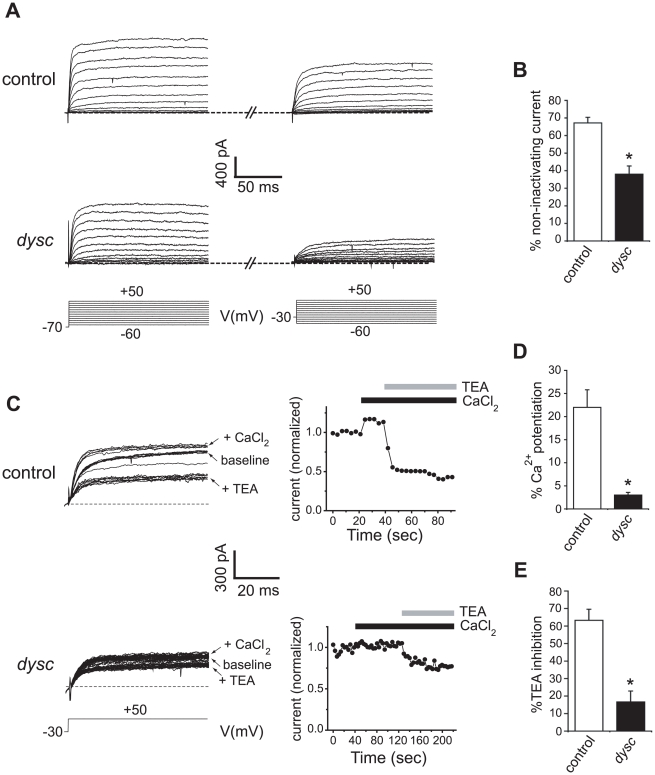
Reduced SLO channel currents in *dysc* mutants. (A) Voltage-dependent outward currents were recorded from *dilp2* neurons in wild-type (upper panels) and *dysc* ^s168^ adult brains (middle panels) in the whole cell patch recording mode. Whole-cell outward currents were evoked by 150 ms depolarizing voltage steps to −60 mV to +50 mV in 10 mV increments (bottom panels), from a holding potential of −70 mV (left) and −30 mV (right) in the same neuron. (B) Quantification of the effect of holding potential in wild-type flies and *dysc* mutants. Control *dilp2* neurons: *N* = 7; *dysc* ^s168^ *dilp2* neurons: *N* = 5. * *p*<0.05, one-tailed Mann-Whitney U-test. (C) Effects of bath application of 2 mM CaCl_2_ and 1 mM TEA on outward currents in wild-type *dilp2* neurons (upper panels) and *dysc* ^s168^ *dilp2* neurons (lower panels). Outward currents were generated by repetitive pulses to a single voltage, +50 mV, from the holding potential of −30 mV (left). Peak current amplitudes plotted against time are also shown (right). Black and gray bars indicate application of CaCl_2_ and TEA, respectively. (D–E) Quantification of the changes in current elicited by addition of 2 mM CaCl_2_ (D) or 1 mM TEA (E) in control and *dysc* ^s168^ *dilp2* neurons. Application of 2 mM CaCl_2_ induces a smaller increase in outward currents in *dysc* ^s168^ *dilp2* neurons relative to wild-type (D). The extent of inhibition by TEA is also far lower in *dysc* ^s168^ *dilp2* neurons (E). Control *dilp2* neurons: *N* = 4; *dysc* ^s168^ *dilp2* neurons: *N* = 3. Error bars represent SEM. * *p*<0.05, one-tailed Mann-Whitney U-test.

To assess the cellular consequences of the regulation of SLO by DYSC, we performed *in vivo* whole-cell patch-clamp electrophysiology on adult *dilp2*-positive neurons in wild-type and *dysc* ^s168^ adult brains. These neurons are located in the pars intercerebralis (PI) and have previously been shown to express a SLO-dependent Ca^2+^-activated non-inactivating potassium current [39]. We chose these neurons for their easy accessibility, and because neurons in the PI were positively labeled by the *dysc*-*Gal4* driver (Figure S4A). Voltage-dependent outward potassium currents were evoked by depolarizing voltage steps in the whole-cell recording mode (Figure 5A). To examine the proportion of non-inactivating potassium component (which includes SLO channels) in the total outward current, we initially applied voltage pulses to pulse potentials ranging from −60 mV to +50 mV from a holding potential of −70 mV. Subsequently, outward currents from the same neuron were evoked via a similar protocol but from a holding potential of −30 mV. Inactivating channels are predominantly inactivated when the membrane potential is held at −30 mV, and non-inactivating channels (including SLO) can thus be isolated from the total outward current. We observed that the outward current at +50 mV in wild-type neurons showed a moderate reduction when the membrane potential was held at −30 mV relative to −70 mV (Figure 5A–5B); in *dysc* mutants, the outward current showed a much greater reduction when the membrane potential was held at −30 mV (Figure 5A–5B), indicating a loss of non-inactivating currents in *dysc* mutants.

To determine what proportion of the non-inactivating outward current is carried by SLO potassium currents, we examined the effect of extracellular Ca^2+^ on outward currents in *dilp2*-neurons, since the SLO channel is highly activated by intracellular Ca^2+^ that enters through Ca^2+^ channels. In wild-type neurons, adding 2 mM CaCl_2_ significantly potentiated the non-inactivating component of the current (Figure 5C–5D). In contrast, the non-inactivating currents in *dysc dilp2*-neurons exhibited only a slight increase upon addition of CaCl_2_. Furthermore, while the application of 1 mM tetraethylammonium (TEA), a blocker of SLO channels [40], reduced the total outward current by 63% in wild-type neurons, it produced only a 17% reduction in *dysc* neurons (Figure 5C and 5E). Thus, the non-inactivating outward current in *dilp2*-neurons is predominantly carried by SLO channels, and is markedly reduced in *dysc* mutants. These results are in accord with our data showing greatly reduced SLO channel expression in *dysc* mutants (Figure 4).

### DYSC and SLO Form a Mutually Dependent Complex

**Figure 6:**
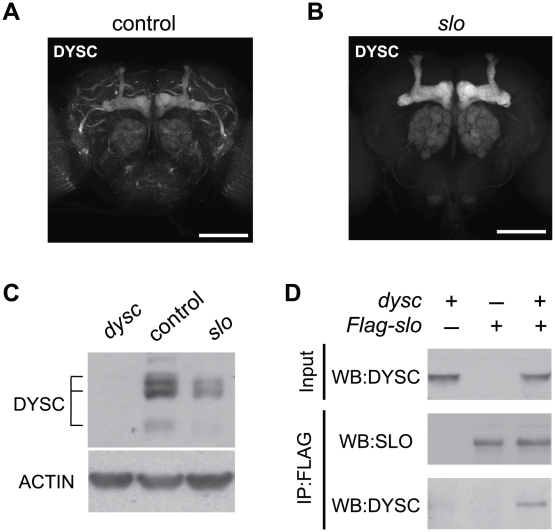
SLO and DYSC form a mutually dependent complex. (A–B) Maximal intensity confocal projections showing DYSC expression in wild-type control (A) and *slo* ^4^ (B) adult brains. Relative to control flies, DYSC expression in neuronal tracts is severely reduced in *slo* ^4^ mutants. In contrast, DYSC expression in the mushroom body lobes, antennal lobes and ellipsoid body remains intact in *slo* ^4^ mutants. Scale bar represents 100 µm. (C) Loss of SLO reduces DYSC expression in head extracts. Both long and short isoforms of DYSC show a clear reduction in *slo* ^4^ mutants. Actin was used as a loading control. (D) DYSC physically interacts with SLO in HEK-tsA cells. Cultured cells were transfected with constructs encoding DYSC and FLAG-tagged SLO, or either construct alone. Cell extracts were immuno-precipitated (IP) using an anti-FLAG antibody, and subjected to western blotting (WB) using an anti-DYSC or anti-SLO antibody.

Finally, we asked if DYSC and SLO exhibit a mutually dependent relationship, since such co-dependence has been demonstrated between Whirlin and its binding partners VLGR1 and Usherin [27], [36], [37]. Interestingly, DYSC in neuronal tracts was largely undetectable in *slo* ^4^ mutants, yet DYSC expression in the mushroom body, ellipsoid body and antennal lobes, areas that do not robustly express SLO, remained intact (Figure 6A–6B). In fact, we noted a significant increase in DYSC levels in the mushroom body lobes in *slo* ^4^ mutants relative to controls (α/β-lobes: increase = 33.1±8%, *p*<0.05, Mann-Whitney U-test; γ-lobes: increase = 41.9±8%, *p*<0.001; controls: *n* = 13 brains, *slo* ^4^: *n* = 10). The mechanistic basis for this increase in DYSC levels is unclear. One possibility is that in the mushroom bodies, DYSC has a mutually dependent relationship with an unidentified protein that is upregulated in the absence of SLO, which leads to an increase in DYSC.

Loss of SLO also resulted in a substantial reduction in total head DYSC protein levels (Figure 6C). SLO and DYSC thus exhibit a reciprocal requirement for expression in neuronal tracts. We did not observe any reduction of *dysc* mRNA levels in *slo* ^4^ mutants, nor any change in *slo* mRNA levels in *dysc* mutants (Figure S8), indicating that the mutual regulation of DYSC and SLO is a post-transcriptional effect.

The reciprocal requirement of SLO and DYSC suggests formation of a stable complex. To examine whether the two proteins can physically interact, we performed co-immunoprecipitation experiments in human embryonic kidney (HEK-tsA) cells. When co-expressed with SLO, DYSC was co-immunoprecipitated with SLO, but not when expressed without SLO (Figure 6D). These data suggest that the two proteins regulate each other's expression through direct protein-protein interaction. In summary, our data identify DYSC as a novel binding partner and regulator of SLO, and suggests that the arrhythmic phenotype exhibited by *dysc* mutants is in part due to a loss of SLO channels.

## Discussion

Here we describe a novel mutant, *dysc*, which exhibits arrhythmic locomotor patterns in DD conditions. Analysis of clock protein oscillations and eclosion rhythms in *dysc* mutants, as well as targeted rescue of the arrhythmic *dysc* phenotype, all indicate that *dysc* is a crucial constituent of the circadian output pathway and acts downstream of the core clock.

One intriguing aspect of *dysc* function is its ontology. Comparative genomics identifies *dysc* as the closest *Drosophila* homolog of *whirlin*, a loci associated with nonsyndromic deafness and Type II Usher syndrome (USH2) in humans [22], [41]. USH is a genetically heterogeneous disorder associated with alterations in cochlear stereocilia structure, vestibular dysfunction and retinitis pigmentosa, resulting in deaf-blindness with varying ages of onset [23]. Our study demonstrates that Usher proteins can also play a crucial role in complex behaviors. This is intriguing given the broad expression of many Usher proteins in the mammalian nervous system, and the lack of functional roles ascribed to the Usher interactome in the brain [29], [30]. Our data also point to a plausible mechanism by which an Usher protein homolog regulates a behavioral output: the control of ion channel expression. This parallels the role of several other PDZ domain-containing proteins in both the mammalian and *Drosophila* nervous systems, such as members of the PSD-95 family, which serve to cluster potassium channels at axons and synapses [42]–[44].

Recent data indicate that the Usher interactome also includes ion channels. Harmonin, a PDZ-containing protein linked to USH1, co-localizes with and negatively regulates the Ca_v_1.3 voltage-gated calcium channel in inner hair cells [38]. We identify a novel interaction between an Usher protein homolog and the SLO Ca^2+^-activated potassium channel. DYSC physically interacts with SLO, and in the absence of DYSC, SLO expression in neuronal tracts as well as SLO currents in *dilp2*-neurons *in vivo* are markedly reduced. DYSC's influence on other potassium channels appears to be limited, since Shaker expression was unaffected in *dysc* mutants, and in *dysc dilp2*-neurons robust outward potassium currents were still detected, albeit with a reduced non-inactivating component caused by the loss of SLO expression. Thus, in contrast to the relationship between Harmonin and Ca_v_1.3 [38], DYSC positively regulates SLO expression in the *Drosophila* brain. Usher proteins and their homologs can therefore both promote and inhibit ion channel function in a subtype-specific manner. It is also noteworthy that both Harmonin's and DYSC's effect on cellular physiology via control of Ca_v_1.3 and SLO respectively is to reduce the excitability of the cell and synaptic output. Thus, one question arising from these studies is whether Usher proteins generally act to negatively tune neuronal excitability. Further studies investigating potential interactions between Usher proteins and other ion channels will help to shed light on this intriguing issue.

We also demonstrate a mutually dependent relationship between DYSC and SLO. In mammals, this finding is paralleled by similar relationships between several Usher proteins and their binding partners [27], [37], [38], and between potassium channels and their associated proteins [45], [46]. Interestingly, whereas loss of SLO greatly reduces DYSC levels in major neuronal tracts in most brain regions, it has an opposite effect in the mushroom body, ellipsoid body, and antennal lobes. In addition, SLO expression is detectable only in the peduncle of the mushroom bodies in *dysc* mutants. These findings raise the possibility that DYSC has a mutually dependent relationship with different proteins depending on the cell type. Given the broad expression in the brain and its interaction with SLO, DYSC is likely to have pleiotropic effects on behavior. SLO is involved in multiple complex behaviors, including the production of courtship songs and ethanol sensitivity [47], [48]. It will be interesting to investigate whether DYSC is also involved in these behaviors.

SLO channels have previously been implicated in the circadian output circuit [7], suggesting a mechanism by which DYSC affects rhythmic behavior. Previous work has demonstrated that loss of SLO de-synchronizes clock protein oscillations in DN clusters [7]. In contrast, in *dysc* mutants the molecular clock is unaffected in these neurons. It is possible that a sufficient level of SLO remains in *dysc* mutants to maintain clock protein cycling. Given our results and the previous finding that restoring SLO in clock cells is not sufficient for a full rescue of the arrhythmic phenotype of *slo* mutants [7], it is likely that SLO performs an important role in the intermediate circuit between the clock and motor neurons where DYSC is required, as well as in clock cells. We propose that DYSC links the central clock output to locomotor activity by regulating membrane excitability, in part through its control of SLO expression. Thus, precise control of neuronal excitability is required not only for correct clock neuron function [49], [50], but also in downstream circuits that connect clock cells to motor neuron targets.

In conclusion, we have identified a novel *Drosophila* ion channel regulator and human disease gene homolog that impacts complex behavior. In addition to shedding new light on genetic components of the circadian output pathway, our results suggest new roles for Whirlin in the mammalian nervous system. It will be interesting to determine if *whirlin* mutants are also arrhythmic, and whether Whirlin similarly regulates SLO expression in the mammalian brain.

## Materials and Methods

### *Drosophila* Strains

Flies were reared on standard food containing cornmeal, yeast, and molasses. The s168 mutant strain was isolated from an ongoing screen for sleep and circadian mutants. Novel strains carrying random insertions of the P[XP] transposable element in a *white* (iso31) background were generated using the Δ2–3 transposase. Sleep and circadian behavior was assayed as previously described [51]. Inverse PCR revealed that the s168 line carries a P-element insertion in the *dysc* locus. Two additional P-element insertion alleles of *dysc*, c05107 and c03838, were obtained from the Exelixis collection at the Harvard Medical School. All three alleles were backcrossed to the iso31 strain at least 5 times, and balanced mutant and sibling control lines were established. The BSC614 deficiency line that removes the *dysc* locus and OK371-, c819-, c107-, and c061-*Gal4* lines were obtained from the Bloomington Stock Center. c164-*Gal4* was obtained from L. Griffith (Brandeis University). Other drivers and the *Shaker* deficiency line were obtained as previously described [46], [52].

Precise excision lines were derived from the s168 line by a transposase-mediated mobilization of the P element. We identified three precise excision lines by PCR amplification and sequencing. Preliminary results indicated that they had similar circadian behavior, and data from one of them are presented. We screened ∼150 excision lines, but were unable to obtain imprecise excision lines that remove coding regions.

### Behavioral Assays

To monitor circadian locomotor behavior, 2- to 5-day old male flies, entrained to a 12 h∶12 h LD cycle for at least 3 days, were put into glass tubes containing 5% sucrose and 2% agar, and their activity was monitored using the *Drosophila* Activity Monitoring System (Trikinetics) at 25°C. For quantification of circadian behavior, activity counts were collected in 30-min bins over a 6-day period in DD. Actograms were generated using ClockLab (Actimetrics), and circadian period and power of rhythmicity were determined using Fly Activity Analysis Suite for Mac OSX (FaasX, M. Boudinot). The power of rhythmicity is defined as the difference between the χ*^2^* value and the significance value at *p* = 0.05. Flies with power of less than 25 were considered arrhythmic, between 25 and 50, weakly rhythmic, and over 50, rhythmic. Circadian period was determined for rhythmic flies only, whereas power of rhythmicity was determined for all flies, including arrhythmic and weakly rhythmic ones. Locomotor patterns in 12 h∶12 h LD conditions were calculated as follows: single-fly activity was monitored over a three day period and averaged to generate a mean 24 h activity plot. This activity plot was then further averaged across the experimental population. For analysis of eclosion behavior, pupae entrained to a 12 h∶12 h LD cycle throughout development were taped to eclosion monitors (Trikinetics) using double-sided tape. Data were collected in 1-h bins over a 4-day period in DD at 25°C. Climbing assays were performed as described previously [46].

### Transgenic Fly Lines

Fly head mRNA was extracted using the Ultraspec RNA Isolation System (Biotecx) and reverse transcribed using High Capacity cDNA Reverse Transcriptase Kit (Applied Biosystems). To generate the UAS-*dysc* construct, *dysc* cDNA was PCR-amplified in two pieces using two sets of primers: for N-terminus: 5′-CTG AAT TCC CAG CAG TGT AAT GC-3′ and 5′-CGA GAA AGG ATT GCC CAT T-3′; for C-terminus: 5′-CGA TCC GGA CTG ATG ATT G-3′ and 5′-CTG GTA CCG GCA GGG CAA GC-3′. The N- and C-terminus fragments were subcloned into the TOPO TA-cloning vector (Invitrogen), sequenced, and subsequently inserted into the pUAST vector. The cloned cDNA represents a novel isoform, differing slightly from other long isoforms in FlyBase (www.flybase.org) through alternative splicing, and we have designated it Isoform G. The C-terminus of the short isoform (F) was amplified using the following primers: 5′-CGA TCC GGA CTG ATG ATT G-3′ and 5′-CTG GTA CCT AAG TGT ATA TAG TGT CTG-3′. To generate the *dysc*-*Gal4* construct, approximately 4.5 Kb upstream of the transcriptional start site was PCR-amplified from genomic DNA using the following primers: 5′-TCC TGC CTC TGG ATC CCG CCA CGT TG-3′ and 5′-ACG CGG CCG CGG CTT CAA ACC AAA TCA GC-3′. The PCR fragment was inserted into the pPT-Gal vector. Transgenic fly lines carrying the UAS*-dysc* or *dysc-Gal4* construct were generated by standard germline transformation in the iso31 background (Rainbow Transgenics).

### Antibody Production and Western Blot Analysis

The rat polyclonal antibody to DYSC (TJR43) was raised against a portion of the DYSC protein fused to N- and C-terminal 6× HIS tags. A PCR fragment amplified using the primers 5′-CCG AAT TCT GCA CCT CCA TCG A-3′ and 5′-CCT TCG ATA GCA ATA CCT CGA GTT-3′ was inserted into the pET-28a vector. Protein expression and purification was performed at the Protein Expression Facility of Wistar Institute. The antibody recognizes both long and short isoforms of DYSC. The rabbit polyclonal antibody to SLO (763) was a generous gift from I. Levitan, and will be described elsewhere. While the antibody detected SLO-specific signal in immuno-staining assays, it could not detect SLO on Western blots due to masking by a non-specific band present in *slo^4^* mutants.

Western blot experiments were carried out essentially as described [46] except that fly heads were homogenized and lysed in 2× SDS sample buffer containing 5% β-mercaptoethanol. Antibodies to PER (PA1139), DYSC (TJR43), SLO (763), and Shaker (UPR55) [46] were used at 1∶1000. Antibodies to MAPK (Sigma) and β-ACTIN (Abcam) were used at 1∶10,000. Western blot experiments were repeated at least three times except as noted, and representative blots are shown.

### Immunohistochemistry

To examine cycling of PER and PDF in the central clock cells, young female flies (1–4 days old) were entrained to a 12 h∶12 h LD cycle for at least 3 days, and were collected at indicated times during the second day in DD. Dissected brains were fixed in 4% paraformaldehyde, and incubated overnight in antibodies to PER (UPR34) and PDF (HH74) [24] diluted 1∶1000. PER and PDF levels were judged through visual inspection. For DYSC and SLO staining, male flies were used. Rat anti-DYSC and rabbit anti-SLO antibodies were used at 1∶400 and 1∶1000, respectively. Fluorophore-conjugated secondary antibodies were obtained from Invitrogen. Brains stained with PER and PDF antibodies were imaged with a Leica TCS-SP5 confocal microscope, and those stained with DYSC and SLO antibodies were imaged with an Olympus Fluoview confocal microscope. Samples for comparison were processed at the same time and imaged with the same settings at sub-saturation intensities. At least five brains were examined per condition. To quantify DYSC levels in the mushroom body lobes, average pixel intensities in the α/β- and γ-lobes of the mushroom bodies were determined in each brain hemisphere using Image J. For each pair of lobes, a mean value was calculated, yielding a single value for α/β- and γ-lobes for each brain. Data from paired batches of control and *slo* ^4^ brains were normalized to the mean of the controls for each batch.

### Co-Immunoprecipitation

For co-immunoprecipitation (co-IP) experiments, the coding region of the G isoform of *dysc* was inserted into the pcDNA3 expression vector using standard molecular biology techniques. The O isoform of SLO (www.flybase.org) was tagged with the 3×FLAG epitope via PCR-driven overlap extension, and was cloned into the pcDNA3 vector. HEK-tsA cells were transfected with various combinations of *dysc* and *Flag-slo* constructs (330 ng each) in 60 mm Petri dishes using Effectene (Qiagen). pCDNA3 vector DNA was included in some conditions to make the total amount of DNA equal in all conditions, and pIRES-*GFP* (330 ng) was included in all conditions to monitor transfection efficiency. Co-IP was performed essentially as described [46] except that cells were lysed in extraction buffer containing 50 mM KCl, 10 mM HEPES, 2 mM EDTA, 5 mM Tris at pH 7.5, 1% Triton X-100, 10% glycerol, 10 µg/mL leupeptin, 10 µg/mL aprotinin, 2 µg/mL pepstatin A, 0.5 mM PMSF, 1 mM Na_3_VO_4_, 10 mM r-nitrophenyl phosphate, pH 7.5, and an antibody to FLAG (Sigma) was used.

### Real-Time Reverse-Transcriptase (RT) PCR

cDNAs from fly heads were generated as described above. Real-time RT-PCR was performed using SYBR green (Applied Biosystems) with the following primers: 5′-CGG CAT TTG CGT TAA AGG AG-3′ and 5′-GAG ATG TAG ACG CCT AAG CCT GAG-3′ for *dysc*, and 5′-GTC GTA CGG AAT GCT GTG CA-3′ and 5′-GAG CTG GTG TCC CTG AAT CG-3′ for *slo*. Both sets of primers recognize regions common to all isoforms.

### *In Vivo* Electrophysiology

*Dilp2*-positive neurons were labeled by driving a membrane-tagged GFP (CD8::GFP) using the *dilp2*-*Gal4* driver, expressed in either a wild-type or *dysc* ^s168^ background. For *in vivo* patch recording from PI neurons [33], [39] flies were anesthetized with CO_2_ and glued ventral side down to a glass coverslip. The coverslip was placed in a chamber containing extracellular solution (101 mM NaCl, 3 mM KCl, 4 mM MgCl_2_, 1.25 mM NaH_2_PO_4_, 20.7 mM NaHCO_3_, 5 mM glucose [pH 7.2]) and then the cuticle was peeled off using fine forceps to expose the surface of the brain. The chamber was placed on the stage of an Olympus BX51 fluorescent microscope, and PI neurons were identified by their location and fluorescence. Patch-recording electrodes (WPI) were fire polished, and had resistances from 3 to 4 MΩ when filled with intracellular solution (102 mM K-gluconate, 17 mM NaCl, 2 mM CaCl_2_, 0.5 mM MgCl2, 5 mM EGTA, 10 mM HEPES, pH 7.2). Standard techniques were used to record macroscopic currents in the whole-cell voltage-clamp mode with an Axopatch 200A amplifier (Molecular Devices). Data were digitized with a Digidata 1322A interface (Molecular Devices) and stored on a PC hard drive for further analysis with pClamp9 software (Molecular Devices).

### Statistical Analysis

For comparison of rhythm strength between pairs of conditions, Student's *t*-tests (unpaired, two-tailed) were performed with Bonferroni correction for multiple comparisons. When comparing multiple experimental genotypes to controls, one-way ANOVA with Dunnett post-hoc tests were used. For electrophysiology data, Mann-Whitney U-tests were performed. Significance values were calculated using Kaleidograph (Synergy Software) or Excel (Microsoft).

## Supporting Information

Figure S1 Normal circadian cycling of PER expression in *dysc* mutant flies. Head extracts of wild-type control and *dysc* mutant flies collected at indicated time points in DD were examined by Western blotting. PER expression levels and phosphorylation exhibited daily oscillations in *dysc* mutants similar to those seen in control flies. MAPK bands were used as a loading control. Similar results were obtained in three independent experiments.
(PDF)

Figure S2 Behavioral assays for locomotor patterns in LD and climbing. (A) Mean activity counts per 30 min were calculated over 3 days in 12 h light (white bars)∶ 12 h dark (black bars) conditions for control and *dysc* males. Black and white arrows indicate the presence and absence of anticipatory increases in activity preceding light-dark transitions, respectively. *N*≥29. (B) Total activity counts during the day and night for the flies shown in (A). (C) Percent of control and *dysc* flies that climb 7 cm in 10 seconds is shown. *N*≥34. ** *p*<0.001, one-way ANOVA with Dunnett post-hoc test. Error bars represent SEM.
(PDF)

Figure S3 Expression of UAS-*dysc* transgenes. Head extracts of wild-type control flies or *dysc* ^c03838^ mutants carrying the *elav*-Gal4 driver or a UAS-*dysc* transgene or both were examined by Western blotting. Both *dysc* transgenes encoding a long (g) or short (f) isoform yielded abundant protein expression. Similar results were obtained in two independent experiments. MAPK was used as a loading control.
(PDF)

Figure S4 Expression patterns of *dysc*-*Gal4* in the adult brain. (A) *dysc*-*Gal4* was used to drive a membrane-bound GFP (mCD8::GFP). A maximum-intensity projection of 2 µm confocal sections spanning the anterior half of the brain is shown. *dysc*-*Gal4* exhibited a broad expression pattern in the central brain. Scale bar, 20 µm. (B) Flies expressing a nuclear GFP under control of *dysc*-*Gal4* were examined for PER and GFP by immuno-staining at ZT2. All small and large LN_v_ clock cells, as well as subsets of the LN_d_ and DN1 neurons, identified by PER nuclear staining, were positive for GFP. Arrowheads indicate GFP- and PER-positive cells. Scale bar, 20 µm.
(PDF)

Figure S5 DYSC protein levels do not show circadian cycling. (A) Head extracts of wild-type control flies collected at indicated time points in LD were examined by Western blotting. Similar results were obtained in three independent experiments. MAPK was used as a loading control. (B) DSYC expression in adult brains at various time points in LD. No apparent change in DYSC expression or localization as a function of time was observed within the brain.
(PDF)

Figure S6 Expression patterns of c164-*Gal4* in the adult brain. (A) c164-*Gal4* was used to drive a membrane-bound GFP (mCD8::GFP). Left panel: maximum-intensity projections of 2 µm confocal sections spanning the anterior half of the brain; right panel: single confocal section illustrating the absence of GFP expression in the ellipsoid body of c164-*Gal4*>GFP males. Arrowhead points to the GFP-negative ellipsoid body. Scale bar, 20 µm. (B) Co-labeling of PER and GFP at ZT2 in c164-*Gal4*>GFP males. A small number of clock cells, identified by PER nuclear staining, were positive for GFP. The number and identity of the clock cells expressing GFP varied. Arrowheads indicate GFP- and PER-positive cells. Scale bars, 20 µm.
(PDF)

Figure S7 DYSC and SLO exhibit partially overlapping patterns of expression. While DYSC and SLO expression clearly overlaps in neuronal tracts in the brain (strong regions of co-localization are indicated by asterisks), DYSC also exhibits relatively robust diffuse expression within the antennal lobes (AL), mushroom body (MB) (upper panel) and the ellipsoid body (EB, lower panel). SLO expression in these regions is relatively weak compared to DYSC.
(PDF)

Figure S8 Transcription of *dysc* and *slo* is unaltered in *slo* ^4^ and *dysc* ^c03838^ mutants, respectively. (A) *slo* transcription in control and *dysc* ^c03838^ heads (*N* = 4). Values were normalized to levels of a control transcript, *Rpl32*. (B) *dysc* transcription in control and *slo* ^4^ heads (*N* = 3). Error bars represent SEM.
(PDF)
