# Supplementary figures and images for: dyschronic, a Drosophila Homolog of a Deaf-Blindness Gene, Regulates Circadian Output and Slowpoke Channels
Source: PLoS Genet. 2012 Apr 19;8(4):e1002671. doi: 10.1371/journal.pgen.1002671 (PMC3330124; doi:10.1371/journal.pgen.1002671)

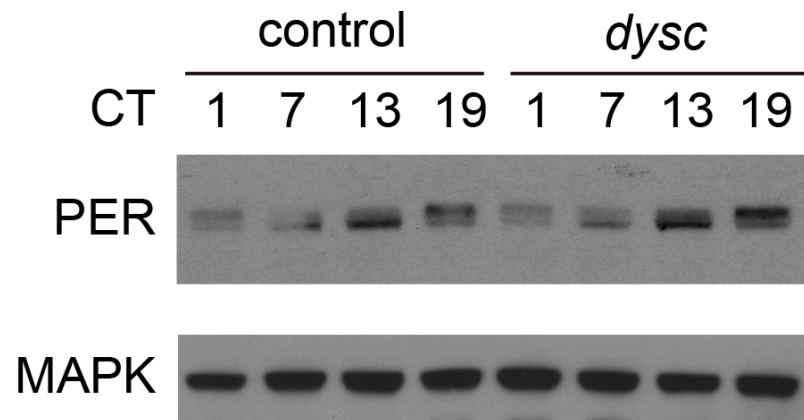

Figure S1

Supplement: Figure S1 — Normal circadian cycling of PER expression in dysc mutant flies. Head extracts of wild-type control and dysc mutant flies collected at indicated time points in DD were examined by Western blotting. PER expression levels and phosphorylation exhibited daily oscillations in dysc mutants similar to those seen in control flies. MAPK bands were used as a loading control. Similar results were obtained in three independent experiments. (PDF) [file pgen.1002671.s001.pdf]

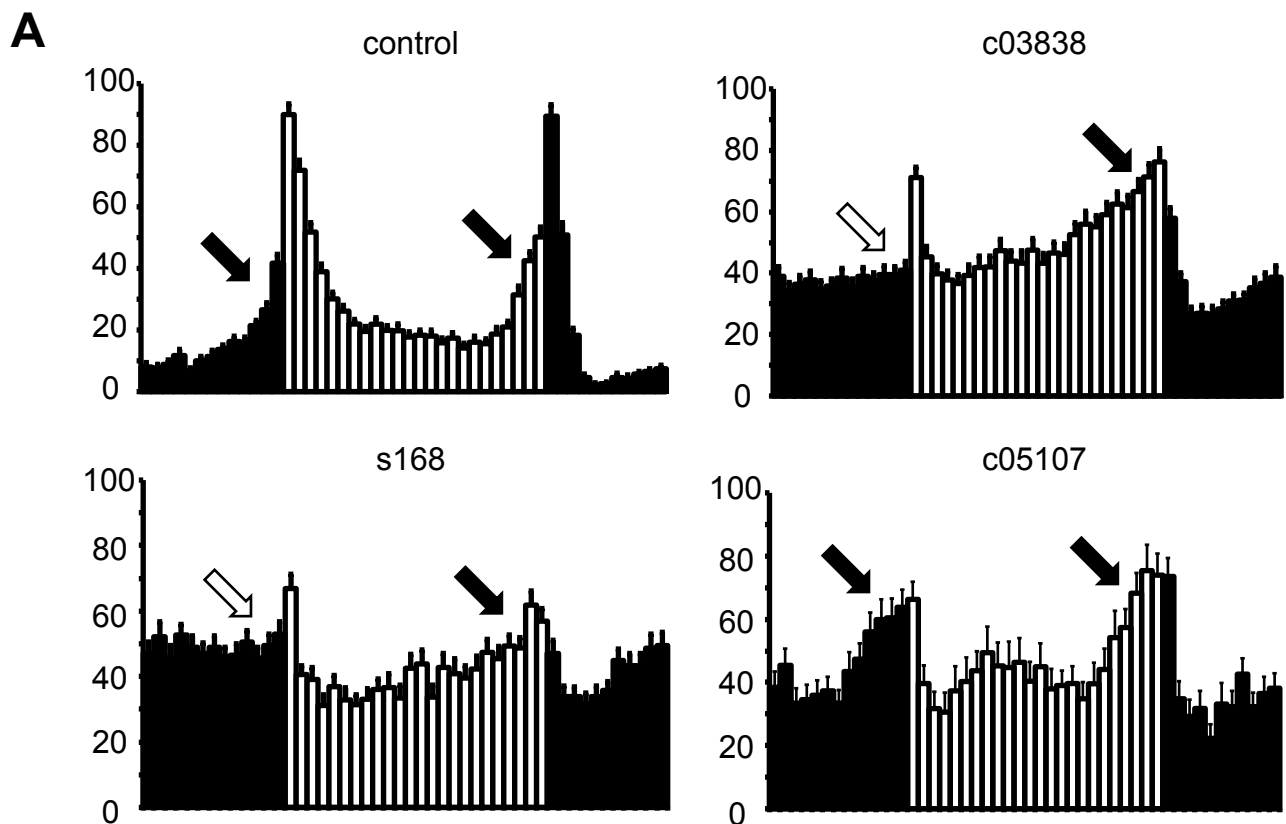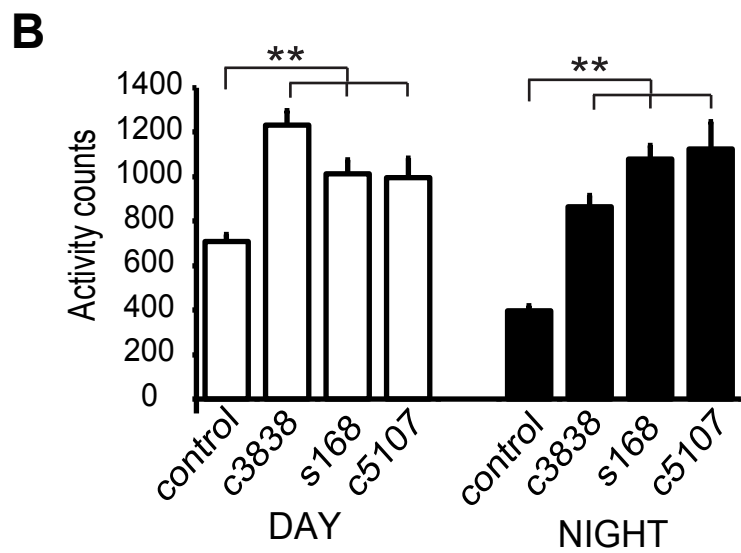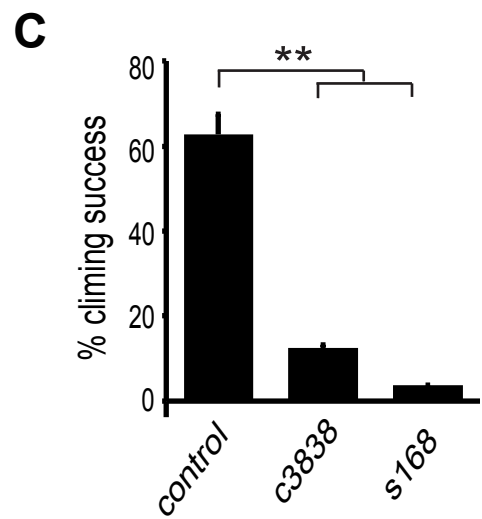

Figure S2

Supplement: Figure S2 — Behavioral assays for locomotor patterns in LD and climbing. (A) Mean activity counts per 30 min were calculated over 3 days in 12 h light (white bars)∶ 12 h dark (black bars) conditions for control and dysc males. Black and white arrows indicate the presence and absence of anticipatory increases in activity preceding light-dark transitions, respectively. N≥29. (B) Total activity counts during the day and night for the flies shown in (A). (C) Percent of control and dysc flies that climb 7 cm in 10 seconds is shown. N≥34. ** p<0.001, one-way ANOVA with Dunnett post-hoc test. Error bars represent SEM. (PDF) [file pgen.1002671.s002.pdf]

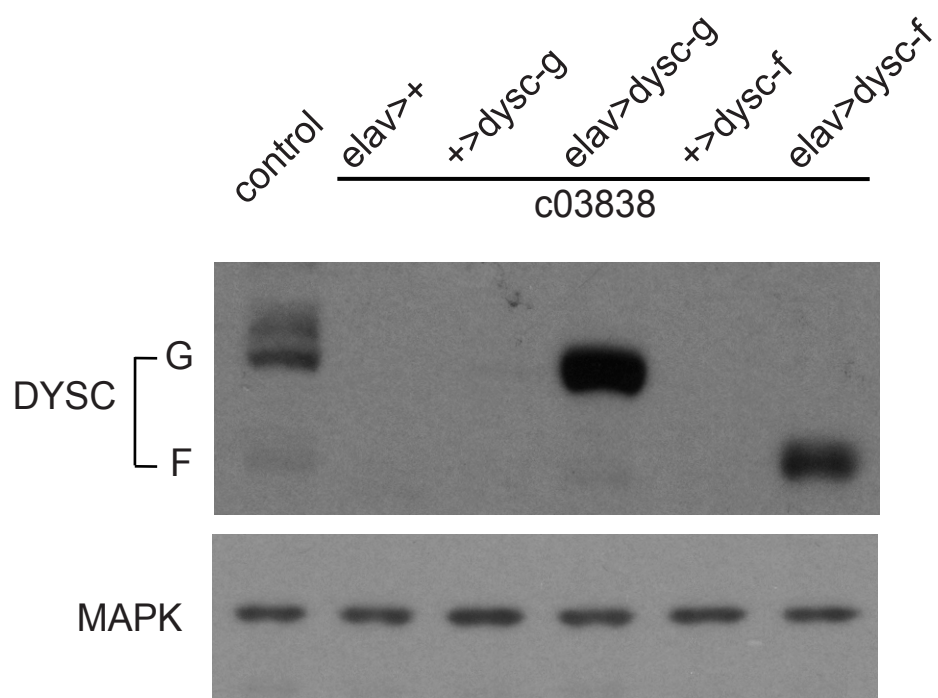

Figure S3

Supplement: Figure S3 — Expression of UAS-dysc transgenes. Head extracts of wild-type control flies or dysc c03838 mutants carrying the elav-Gal4 driver or a UAS-dysc transgene or both were examined by Western blotting. Both dysc transgenes encoding a long (g) or short (f) isoform yielded abundant protein expression. Similar results were obtained in two independent experiments. MAPK was used as a loading control. (PDF) [file pgen.1002671.s003.pdf]

**A**

dysc-Gal4 &gt; GFP

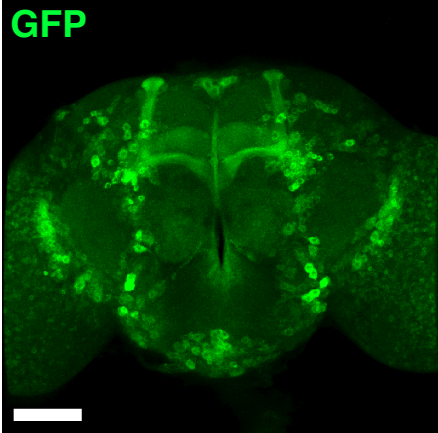**B**

LNvs

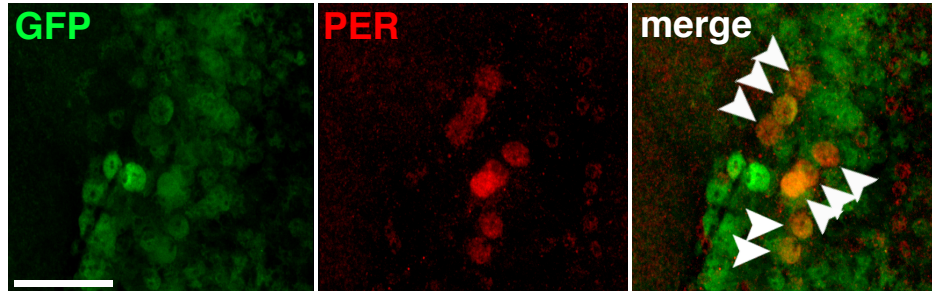

LNds

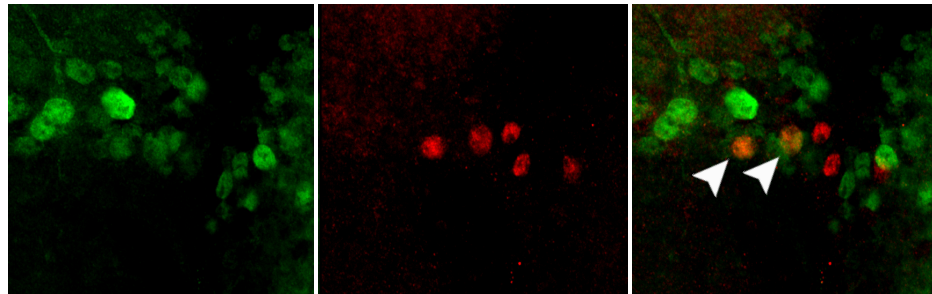

DN1

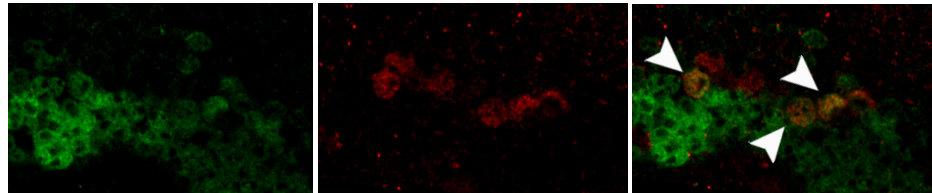

Figure S4

Supplement: Figure S4 — Expression patterns of dysc-Gal4 in the adult brain. (A) dysc-Gal4 was used to drive a membrane-bound GFP (mCD8::GFP). A maximum-intensity projection of 2 µm confocal sections spanning the anterior half of the brain is shown. dysc-Gal4 exhibited a broad expression pattern in the central brain. Scale bar, 20 µm. (B) Flies expressing a nuclear GFP under control of dysc-Gal4 were examined for PER and GFP by immuno-staining at ZT2. All small and large LNv clock cells, as well as subsets of the LNd and DN1 neurons, identified by PER nuclear staining, were positive for GFP. Arrowheads indicate GFP- and PER-positive cells. Scale bar, 20 µm. (PDF) [file pgen.1002671.s004.pdf]

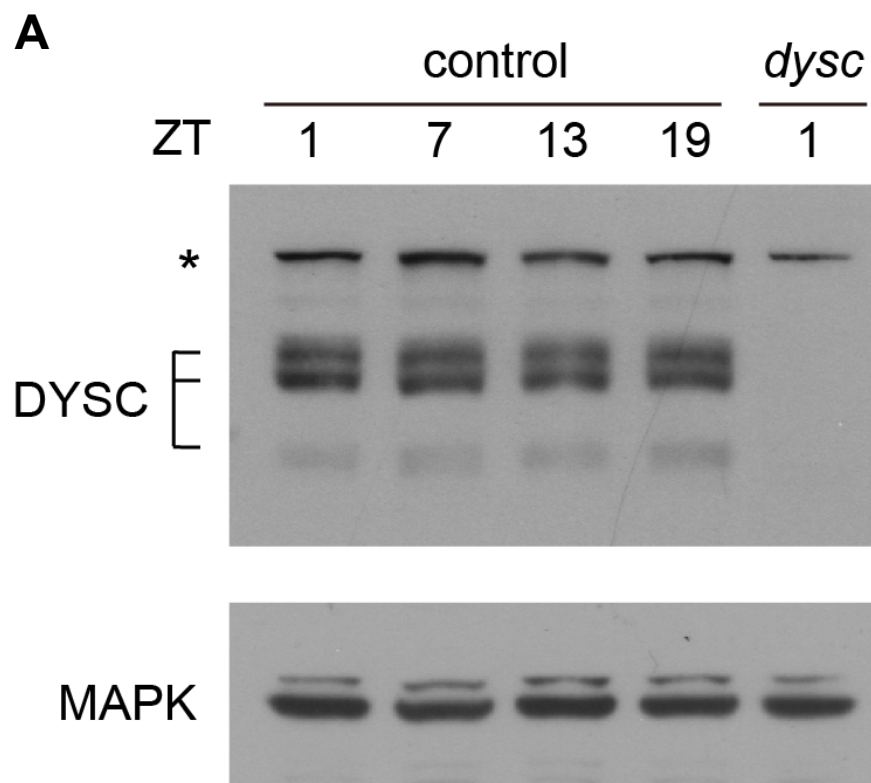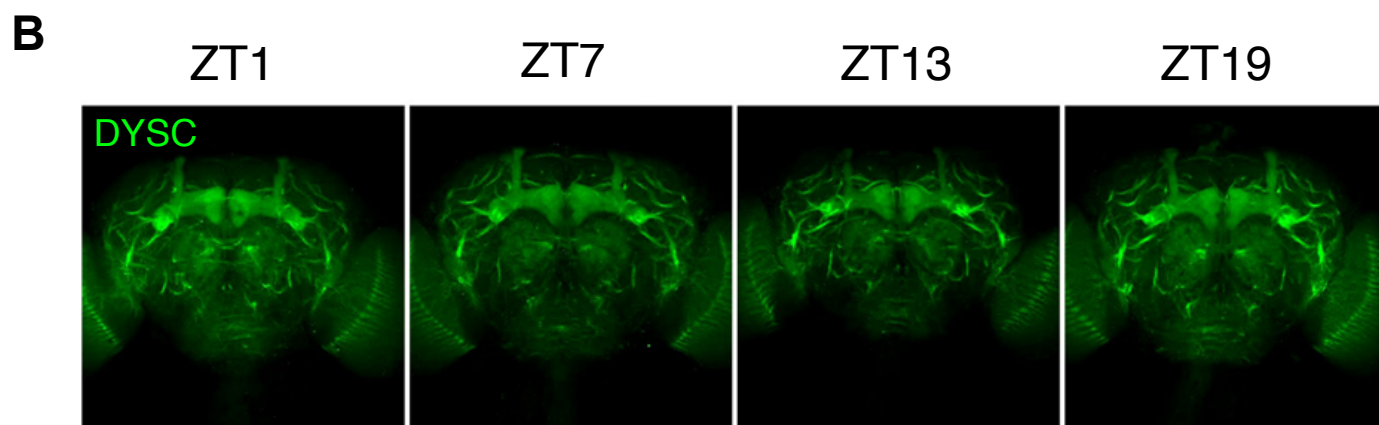

Figure S5

Supplement: Figure S5 — DYSC protein levels do not show circadian cycling. (A) Head extracts of wild-type control flies collected at indicated time points in LD were examined by Western blotting. Similar results were obtained in three independent experiments. MAPK was used as a loading control. (B) DSYC expression in adult brains at various time points in LD. No apparent change in DYSC expression or localization as a function of time was observed within the brain. (PDF) [file pgen.1002671.s005.pdf]

**A***c164-Gal4 > GFP*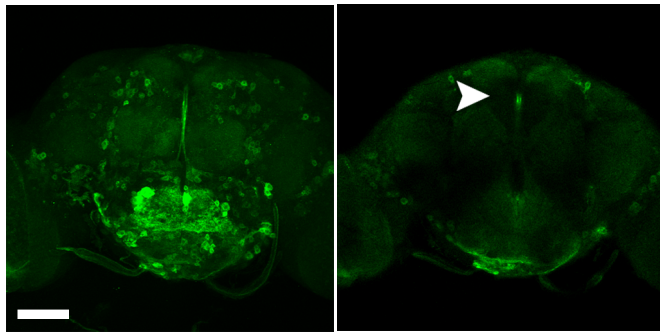**B**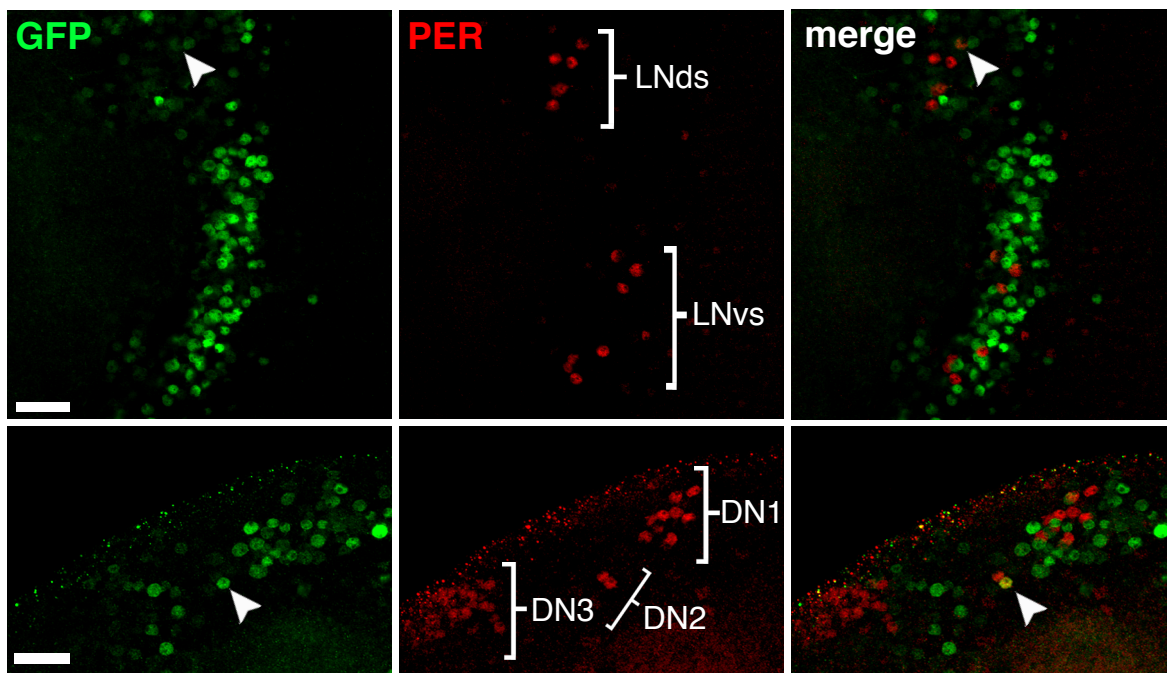

Figure S6

Supplement: Figure S6 — Expression patterns of c164-Gal4 in the adult brain. (A) c164-Gal4 was used to drive a membrane-bound GFP (mCD8::GFP). Left panel: maximum-intensity projections of 2 µm confocal sections spanning the anterior half of the brain; right panel: single confocal section illustrating the absence of GFP expression in the ellipsoid body of c164-Gal4>GFP males. Arrowhead points to the GFP-negative ellipsoid body. Scale bar, 20 µm. (B) Co-labeling of PER and GFP at ZT2 in c164-Gal4>GFP males. A small number of clock cells, identified by PER nuclear staining, were positive for GFP. The number and identity of the clock cells expressing GFP varied. Arrowheads indicate GFP- and PER-positive cells. Scale bars, 20 µm. (PDF) [file pgen.1002671.s006.pdf]

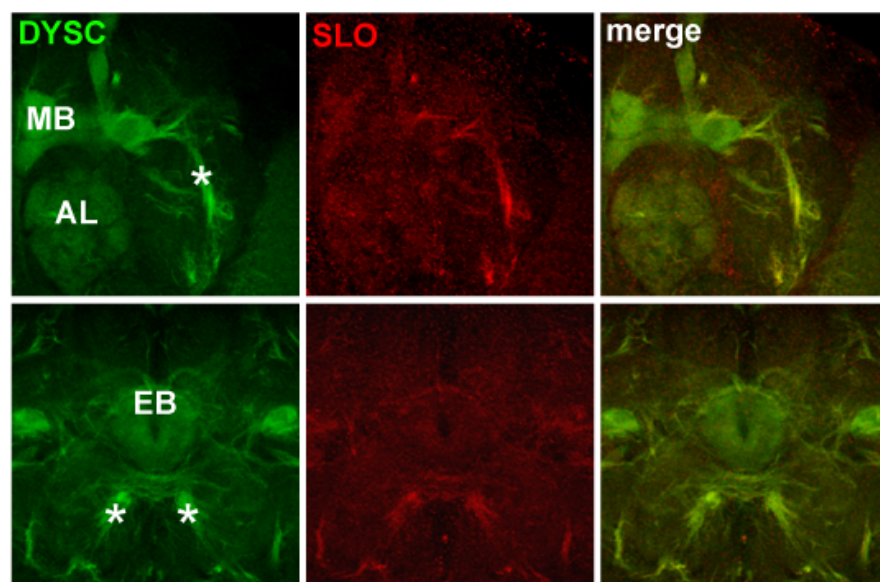

Figure S7

Supplement: Figure S7 — DYSC and SLO exhibit partially overlapping patterns of expression. While DYSC and SLO expression clearly overlaps in neuronal tracts in the brain (strong regions of co-localization are indicated by asterisks), DYSC also exhibits relatively robust diffuse expression within the antennal lobes (AL), mushroom body (MB) (upper panel) and the ellipsoid body (EB, lower panel). SLO expression in these regions is relatively weak compared to DYSC. (PDF) [file pgen.1002671.s007.pdf]

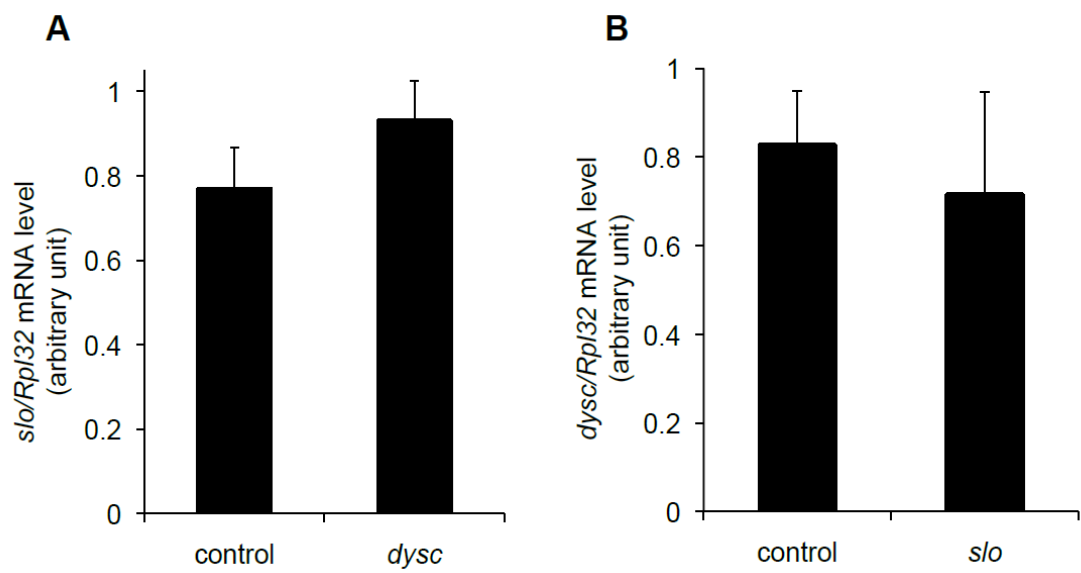

**Figure S8**

Supplement: Figure S8 — Transcription of dysc and slo is unaltered in slo 4 and dysc c03838 mutants, respectively. (A) slo transcription in control and dysc c03838 heads (N = 4). Values were normalized to levels of a control transcript, Rpl32. (B) dysc transcription in control and slo 4 heads (N = 3). Error bars represent SEM. (PDF) [file pgen.1002671.s008.pdf]
